# Clonal Complex 398 Methicillin-Resistant *Staphylococcus aureus* Producing Panton-Valentine Leukocidin, Czech Republic, 2023

**DOI:** 10.3201/eid3101.241323

**Published:** 2025-01

**Authors:** Kristýna Brodíková, Thibault Destanque, Marisa Haenni, Renáta Karpíšková

**Affiliations:** Masaryk University, Brno, Czech Republic (K. Brodíková, R. Karpíšková); ANSES–Université de Lyon, Lyon, France (T. Destanque, M. Haenni)

**Keywords:** Methicillin-resistant *Staphylococcus aureus*, MRSA, CC398, antimicrobial resistance, bacteria, staphylococci, virulence, molecular characterization, Panton-Valentine leucocidin, PVL, ST1232, SNP-based phylogeny, Tn*554*, *ermA*, Czech Republic

## Abstract

To trace evolution of Panton-Valentine leucocidin–positive clonal complex 398 methicillin-resistant *Staphylococcus aureus* (MRSA) in the Czech Republic, we tested 103 MRSA isolates from humans. Five (4.9%) were Panton-Valentine leucocidin–positive clonal complex 398, sequence types 1232 and 9181. Spread to the Czech Republic may result from travel to or from other countries.

Methicillin-resistant *Staphylococcus aureus* (MRSA) clonal complex (CC) 398 was initially found in animals but has since adapted to humans. Human-adapted variants display tetracycline resistance, typically through the *tetK* gene, and possess virulence genes that enable human-to-human transmission (*1*). Some strains also produce Panton-Valentine leukocidin (PVL), a toxin absent in animal-associated CC398. Since 2005, PVL-positive CC398 MRSA has been reported in East Asia and Europe (*2*,*3*). Early cases in Europe were recorded in Sweden and the Netherlands, followed by outbreaks in Denmark (*3*–*5*). With this study, we detected and characterized PVL-positive CC398 MRSA in the Czech Republic and compared our findings with international data to trace the evolution of those strains.

## The Study

During 2021–2023, we obtained MRSA isolates from 2 clinical laboratories in 2 regions of the Czech Republic. We included in our study only unique-patient isolates for that period. All 103 MRSA isolates had limited patient information (specimen type and region) and were confirmed as *mecA*-MRSA by PCR targeting the SA-442 species-specific fragment and the *mecA* gene (*6*,*7*). We assessed CC398 affiliation and presence of the *lukF/lukS-PV* gene by using PCR (*8*,*9*). We performed phenotypic detection of antibiotic resistance by using the disk-diffusion method and interpreted the results according to guidelines provided by the European Committee on Antimicrobial Susceptibility Testing version 14.0 (*10*). We extracted DNA by using the NucleoSpin Microbial DNA isolation kit (Machery-Nagel, https://www.mn-net.com). Library preparation and whole-genome sequencing were outsourced to Eurofins (Stade, Germany), where Illumina NovaSeq6000 technology (https://www.illumina.com) was used. Reads were quality trimmed and de novo assembled by using Shovill v1.0.4 (https://github.com/tseemann/shovill), and we assessed assembly quality by using QUAST v5.0.2 (https://quast.sourceforge.net).

We performed typing by using MLSTFinder v2.0.9 and spaTyper (Genomic Epidemiology Center, http://www.genomicepidemiology.org) and identified resistance and virulence genes by using ResFinder 4.1 and VirulenceFinder v2.0.3 (Genomic Epidemiology Center) (identity ˃95%) and confirmed resistance genes by using CARD 3.2.9. (https://card.mcmaster.ca). We characterized the genetic environment of transposon Tn*554* by using Bakta 1.9.1 (https://bakta.computational.bio). To compare sequences, we used the National Center for Biotechnology Information (NCBI) BLASTn tool (https://blast.ncbi.nlm.nih.gov).

We constructed a single-nucleotide polymorphism–based phylogeny by using Roary as previously published (*6*) (Roary v3.13.0, Gubbins v2.4.1, and snp-dists v0.7.0; https://github.com) on all CC398 PVL-positive isolates retrieved from the RefSeq database (https://www.ncbi.nlm.nih.gov/refseq) as of March 2024 and from selected publications of interest from which data were not retrieved in the RefSeq database (*1*,*4*,*5*,*11*,*12*). We used iTOL v6 (https://itol.embl.de) to visualize phylogenetic trees. Raw data of the sequenced strains are available in GenBank (accession no. PRJNA1095719). 

We tested 103 human MRSA isolates from the Czech Republic; 5 (4.9%) isolates were identified as CC398 and PVL positive and 8 (7.8%) as CC398 and PVL negative. All 5 PVL-positive isolates came from abscess swab samples; the average patient age was 27 years (range 18–45 years). Three patients were from outside the Czech Republic (2 from Asia, 1 from Ukraine), and the other 2 were Czech nationals with no known travel history. All 5 isolates showed identical phenotypes: resistant to cefoxitin, clindamycin, erythromycin, and tetracycline. Four isolates belonged to ST1232 with *spa*-type t034, and the fifth isolate was ST9181 (a single-locus variant of ST1232) with *spa*-type t571. *spa*-types t571 and t034 are closely related. All isolates carried the SCC*mec* type V(5C2).

We analyzed the *ermA* and *ant (**9**)-Ia* genes as part of the Tn*554* transposon on the PVL-positive isolates, which includes transposition-related genes *tnpA*, *tnpB*, and *tnpC* with the resistance genes oriented in opposite directions (Figure). We identified the *ermA* gene by using ResFinder (sequence identity 95%) and CARD (sequence identity 85%). Alignment with the ResFinder reference (EU348758) revealed 21 nt differences compared with the reference from *Streptococcus suis*. BLASTn and BLASTp analyses showed 100% identity/coverage at nucleotide and protein levels for *ermA* variants in PVL-positive ST1232 genomes, explaining the undetected *ermA* gene in some MRSA ST1232 studies despite reported phenotypic resistance (Appendix).

**Figure:**
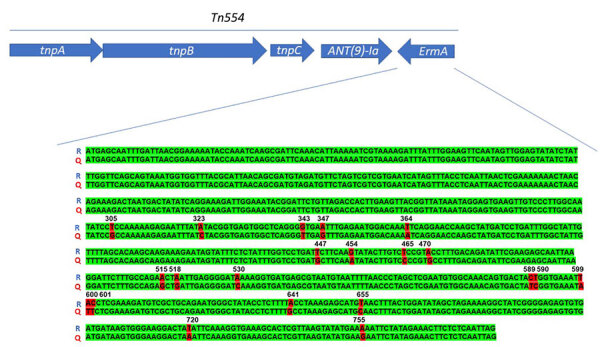
Schematic representation of the Tn*544* transposon carrying the *ermA* and *ant(**9**)-Ia* genes representative of methicillin-resistant *Staphylococcus aureus* Panton-Valentine–positive clonal complex 398. The *ermA* gene sequence of the strains isolated from patients in the Czech Republic in 2023 align with the reference sequence of the ResFinder software (Genomic Epidemiology Center, http://www.genomicepidemiology.org). The resistance genes *ermA* and *ant(**9**)-Ia* (arrows) are transcribed in different directions because they are located on different strands of the DNA. Q, query sequence; R, reference sequence; tnpA, transposase A; tnpB, transposase B; tnpC, transposase C.

We constructed a single-nucleotide polymorphism–based phylogenetic tree (Appendix Figure), incorporating PVL-positive isolates from the RefSeq database and major publications. We also included PVL-negative human and animal genomes from clades I, II-GOI, and IIa-GOI, positioning the 5 isolates from the Czech Republic within evolutionary pathways of related isolates. The ST1232 genomes, including those from the Czech Republic, formed a subclade within II-GOI, emerging since 2013 as the main PVL-positive CC398 carriers.

## Conclusions

MRSA CC398 is commonly associated with livestock, characterized by tetracycline resistance (*tetM*) and lacking virulence factors such as immune evasion cluster and PVL. Human-origin MRSA often carries immune evasion cluster and PVL genes. We found 4.9% PVL-positive CC398 isolates in the Czech Republic. However, the limited scope of our study (103 isolates from 2 regions) suggests the need for a nationwide survey to better assess PVL-positive CC398 dissemination. A larger-scale study could determine if PVL-positive ST1232 is particularly associated with abscesses in younger patients, as seen in our study (average patient age 27 years). Various studies suggest that those strains can cause infections in younger populations, as evidenced in Denmark, where ST1232 infected both mothers and children, and in a Maternity and Children’s Health Care Center in China, where ST1232 was the leading cause of skin and soft tissue infections (*5*,*13*).

The spread of PVL-positive MRSA CC398 strains could also be the result of travel to foreign countries and tourists visiting the Czech Republic. Of the patients that we report, 3 were foreigners: 2 from unspecified countries from eastern Asia and 1 from Ukraine. As early as 2008, links with countries in Asia were noted (*3*).

ST1232 emerged as the new successful subclade, as described by Schouls et al. in a large genomic analysis of 4,991 MRSA strains, which showed that all previous PVL-positive CC398 strains belonged to ST398 and the more recent ones to ST1232 (*12*). That finding is also visible on our phylogenetic tree (Appendix Figure), on which the cluster of ST1232 isolates, which encompassed the 5 isolated from the Czech Republic, emerged as a subclade of the clade II-GOI described by Price et al. (*1*).

Among the PVL-positive isolates, ST1232 differed from ST398 by the quasi-systematic presence of the *tetK* gene and by the chromosomal insertion of the Tn*554* transposon. The *ermA* gene harbored multiple point mutations compared with other *ermA* sequences from *S.* *aureus* found in GenBank or with the reference sequence from an *S. suis* genome, used by ResFinder. 

A literature review revealed that, although all publications reported a macrolide–lincosamide–streptogramin B phenotype using antibiograms or microdilution, few identified the genetic mechanisms of that resistance, especially with PCR. We recommend using the primers from Koike et al. (*14*), which effectively detect the ST1232 *ermA* variant, and, if PCR for *ermA* is negative, checking primer efficiency and supplementing with phenotypic testing. For genomic analysis of whole-genome sequencing data, updating the resistance database would be useful because of additional resistances found in multidrug resistant, virulent PVL-positive CC398 isolates.

In summary, we report the presence of PVL-positive MRSA CC398 in the Czech Republic, accounting for 4.9% of the MRSA isolates. We recommend using whole-genome sequencing to differentiate between human and animal strains, detect erythromycin-resistance genes, and rapidly identify sequence types. Comprehensive knowledge of the genetic and epidemiologic characteristics of ST1232 is essential for developing effective public health strategies against PVL-positive CC398.

Appendix Additional information from study of clonal complex 398 methicillin-resistant *Staphylococcus aureus* producing Panton-Valentine leucocidin, Czech Republic, 2023.
