# Supplementary material for: Clonal Complex 398 Methicillin-Resistant Staphylococcus aureus Producing Panton-Valentine Leukocidin, Czech Republic, 2023
Source: Emerg Infect Dis. 2025 Jan;31(1):174–7. doi: 10.3201/eid3101.241323 (PMC11682819; doi:10.3201/eid3101.241323)
Supplement: Appendix — Additional information from study of clonal complex 398 methicillin-resistant Staphylococcus aureus producing Panton-Valentine leucocidin, Czech Republic, 2023. [file 24-1323-Techapp-s1.pdf]

Article DOI: https://doi.org/10.3201/eid3101.241323

EID cannot ensure accessibility for supplementary materials supplied by authors. Readers who have difficulty accessing supplementary content should contact the authors for assistance.

Clonal Complex 398 Methicillin-Resistant *Staphylococcus aureus* Producing Panton-Valentine Leukocidin, Czech Republic, 2023

Appendix

Appendix Table. List of publications providing information on the ability to detect the *ermA* and *ant (9)-Ia* genes

Country	Year of publication	Type of resistance gene detection	Detection of gene *ermA*	Detection of gene *ant (9)-Ia*	Phenotype resistance to macrolide	Publication
NZ	2014	DNA microarray analysis	yes	-	yes	(*1*)
CN	2018	MIC	-	-	yes	(*2*)
DK	2018	unspecified	yes	yes	yes	(*3*)
DE	2020	Abricate 0.8.13*	**no**	yes	yes	(*4*)
JP	2020	PCR	**no**	-	yes	(*5*)
JP	2021	PCR	**no**	no	yes	(*6*)
JP	2021	MIC	-	-	yes	(*7*)
AU	2022	Resfinder	yes	yes	yes	(*8*)
NL	2023	Resfinder	yes	yes	yes	(*9*)
NL	2023	unspecified	yes	yes	yes	(*10*)
JP	2023	PCR	**no**	-	yes	(*11*)
SK	2023	Resfinder	yes	yes	yes	(*12*)
SK	2024	AMRfinderPlus v.3.10.30	yes	yes	yes	(*13*)

*Includes ResFinder 3.0, ARG-ANNOT, CARD, NCBI-BARRGD.

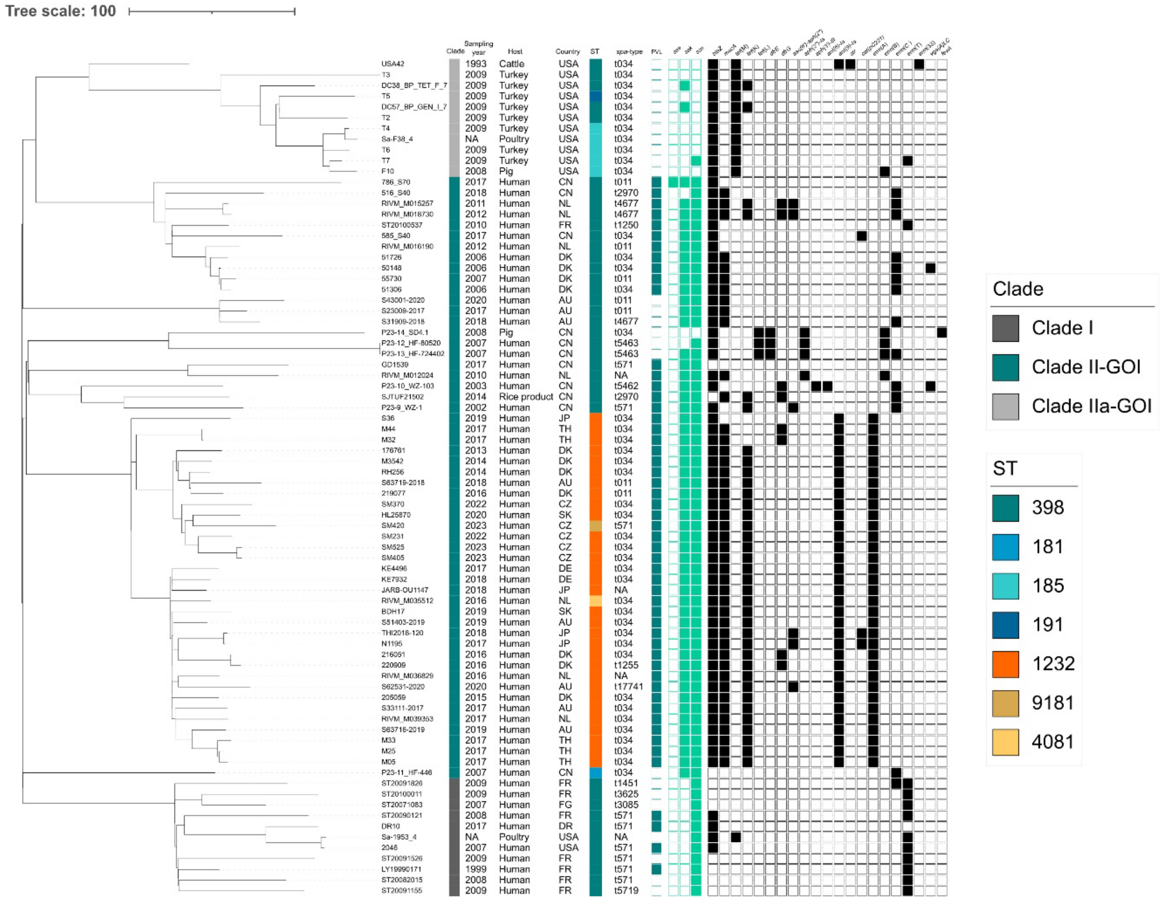

Appendix Figure. Single-nucleotide polymorphism–based phylogeny of methicillin-resistant *Staphylococcus aureus* clonal complex 398 isolates. The 3 I, II-GOI, and IIa-GOI clades are by Price et al. (*2*). AU, Australia; CN, China; CZ, Czech Republic; DE, Germany; DK, Denmark; DR, Dominican Republic; FG, French Guinea; FR France; JP, Japan; NL, Netherlands; SK, South Korea; TH, Thailand.

References

1. Williamson DA, Bakker S, Coombs GW, Tan H, Monecke S, Heffernan H. Emergence and molecular characterization of clonal complex 398 (CC398) methicillin-resistant Staphylococcus aureus (MRSA) in New Zealand. J Antimicrob Chemother. 2014;69:1428–30. PubMed https://doi.org/10.1093/jac/dkt499

2. Liang B, Mai J, Liu Y, Huang Y, Zhong H, Xie Y, et al. Prevalence and characterization of *staphylococcus aureus* isolated from women and children in Guangzhou, China. Front Microbiol. 2018;9:2790. PubMed https://doi.org/10.3389/fmicb.2018.02790

3. Møller JK, Larsen AR, Østergaard C, Møller CH, Kristensen MA, Larsen J. International travel as source of a hospital outbreak with an unusual meticillin-resistant *Staphylococcus aureus* clonal complex 398, Denmark, 2016. Euro Surveill. 2019;24:1800680. PubMed https://doi.org/10.2807/1560-7917.ES.2019.24.42.1800680

4. Klein S, Hannesen J, Zanger P, Heeg K, Boutin S, Nurjadi D. Entry of Panton-Valentine leukocidin-positive methicillin-resistant *Staphylococcus aureus* into the hospital: prevalence and population structure in Heidelberg, Germany 2015-2018. Sci Rep. 2020;10:13243. PubMed https://doi.org/10.1038/s41598-020-70112-z

5. Nakaminami H, Hirai Y, Nishimura H, Takadama S, Noguchi N. Arthritis Caused by MRSA CC398 in a patient without animal contact, Japan. Emerg Infect Dis. 2020;26:795–7. PubMed https://doi.org/10.3201/eid2604.190376

6. Aung MS, Urushibara N, Kawaguchiya M, Hirose M, Ito M, Habadera S, et al. Clonal diversity of methicillin-resistant *Staphylococcus aureus* (MRSA) from bloodstream infections in northern Japan: Identification of spermidine N-acetyltransferase gene (speG) in staphylococcal cassette chromosomes (SCCs) associated with type II and IV SCCmec. J Glob Antimicrob Resist. 2021;24:207–14. PubMed https://doi.org/10.1016/j.jgar.2020.12.008

7. Nakaminami H, Kawasaki H, Takadama S, Kaneko H, Suzuki Y, Maruyama H, et al. Possible Dissemination of a Panton-Valentine leukocidin-positive livestock-associated methicillin-resistant *Staphylococcus aureus* CC398 clone in Tokyo, Japan. Jpn J Infect Dis. 2021;74:82–4. PubMed https://doi.org/10.7883/yoken.JJID.2020.345

8. Coombs GW, Daley D, Shoby P, Yee NWT, Robinson JO, Murray R, et al. Genomic characterisation of CC398 MRSA causing severe disease in Australia. Int J Antimicrob Agents. 2022;59:106577. PubMed https://doi.org/10.1016/j.ijantimicag.2022.106577

9. Schouls LM, Witteveen S, van Santen-Verheuvel M, de Haan A, Landman F, van der Heide H, et al.; Dutch MRSA surveillance study group. Molecular characterization of MRSA collected during national surveillance between 2008 and 2019 in the Netherlands. Commun Med (Lond). 2023;3:123. PubMed https://doi.org/10.1038/s43856-023-00348-z

10. Gooskens J, Konstantinovski MM, Kraakman MEM, Kalpoe JS, van Burgel ND, Claas ECJ, et al. Panton-Valentine leukocidin-positive CC398 MRSA in urban clinical settings, the Netherlands. Emerg Infect Dis. 2023;29:1055–7. PubMed https://doi.org/10.3201/eid2905.221717

11. Kaneko H, Kanai M, Saito T, Yanagi Y, Kobayashi H, Kurihara R, et al. Significant increase in the prevalence of Panton-Valentine leukocidin-positive methicillin-resistant *Staphylococcus aureus*, particularly the USA300 variant ΨUSA300, in the Japanese community. Microbiol Spectr. 2023;11:e0124823. PubMed https://doi.org/10.1128/spectrum.01248-23

12. Lee GY, Park JH, Lim JH, Yang SJ. Complete genome sequences of two clonal complex 398 methicillin-resistant *Staphylococcus aureus* strains isolated from patients in Korea. Microbiol Biotechnol Lett. 2023;51:132–3.

13. Takahashi T, Kim H, Kim HS, Kim HS, Song W, Kim JS. Comparative genomic analysis of staphylococcal cassette chromosome *mec* type V *Staphylococcus aureus* strains and estimation of the emergence of SCC*mec* V clinical isolates in Korea. Ann Lab Med. 2024;44:47–55. PubMed https://doi.org/10.3343/alm.2024.44.1.47
